# The Effects of Cervical Mobilization with Clinical Pilates Exercises on Pain, Muscle Stiffness and Head and Neck Blood Flow in Cervicogenic Headache: Randomized Controlled Trial

**DOI:** 10.3390/medicina60060852

**Published:** 2024-05-23

**Authors:** Meltem Uzun, Mehmet Ali İkidağ, Yasemin Ekmekyapar Fırat, Nevin Ergun, Türkan Akbayrak

**Affiliations:** 1 Department of Physiotherapy and Rehabilitation, Faculty of Health Sciences, SANKO University, 27090 Gaziantep, Turkey; nevinergun53@gmail.com; 2 Department of Radiology, SANKO University Hospital, 27090 Gaziantep, Turkey; mikidag@hotmail.com; 3 Department of Neurology, Faculty of Medicine, SANKO University, 27090 Gaziantep, Turkey; yaseminekmekyapar@gmail.com; 4 Faculty of Physical Therapy and Rehabilitation, Hacettepe University, 06100 Ankara, Turkey; takbayrak@yahoo.com

**Keywords:** cervicogenic headache, neck, musculoskeletal diseases, blood flow, Pilates-based exercises

## Abstract

*Background and Objectives*: Physiotherapy approaches are used to eliminate the problems caused by cervicogenic headache (CHA), known as secondary headache associated with the structures of the upper cervical region. This study aimed to investigate the effects of cervical mobilization (CM) with clinical Pilates exercises (CPE) on pain, muscle stiffness and head–neck blood flow in CGH. *Materials and Methods*: A total of 25 patients participated in this randomized controlled study and were randomized into either the CM group or the CM+CPE group. All treatment methods were applied 3 days a week for 6 weeks. The outcome measure was headache intensity and frequency, the number of analgesics, muscle stiffness and vertebral artery (VA) and internal carotid artery (ICA) blood flow. Headache intensity was measured by a visual analogue scale, muscle stiffness by a myotonometer and blood flow by Doppler US. Evaluations were repeated after 6 weeks of treatment. Within-group comparisons were made by the Wilcoxon signed rank test, and between-group comparisons were made by the Mann–Whitney U test. *Results*: After treatments in the two groups, headache intensity and frequency and the number of analgesics decreased, the muscle stiffness of the suboccipital, upper trapezius and sternocleidomastoid (SCM) muscles decreased, and the blood flow of the ICA and VA increased (*p* < 0.05). There was a significant difference between the groups in terms of headache intensity (*p* = 0.025) and muscle stiffness in SKM (*p* = 0.044) in favor of the CM+CPE group. *Conclusions*: Non-pharmacological treatment approaches have an important role in CHA related to the upper cervical region. This study suggests that it would be beneficial to add CM in combination with CPE to the non-pharmacological treatment of patients with CHA.

## 1. Introduction

A cervicogenic headache (CHA) is a type of headache of musculoskeletal origin, especially associated with the structures of the upper cervical region. CHA is defined in the International Classification of Headache Disorders as one of the secondary headache types spreading from the back of the head and neck to the ear and zygomatic region, with unilateral compressive and sometimes throbbing character. While pain can be triggered after mechanical maneuvers, it can last for weeks, starting from a few days, and can have a significant negative impact on people’s quality of life [1].

It has been reported that cervical mobility decreases in CHA and muscle functions of the cervical region are adversely impacted [2,3]. In particular, the weakness of the deep neck flexors comes to the fore, and there are studies reporting the presence of anterior tilt posture of the head [4,5]. Accordingly, various studies have determined the presence of stiffness in the muscles of the neck region [6]. In headache problems, musculoskeletal changes such as spinal mobility, muscle weakness, muscle stiffness and posture disorders should be evaluated, and appropriate treatment approaches should be applied in light of the results obtained. The importance of a multidisciplinary approach in headaches in terms of patient satisfaction with treatment has been reported in the literature [7].

There are types of exercises that have been reported to reduce muscle stiffness in CHA, but more randomized controlled studies are needed [8,9]. Although studies in the literature are mostly related to primary headache types, there are various studies examining the presence of head and neck blood flow changes in headache [10,11]. However, there are no studies that investigate changes in the blood flow of the arteries feeding the head and neck in physiotherapy approaches applied in CHA. The present study eliminates this deficiency in the literature and is pioneering in this respect.

Manual therapy approaches are recommended in studies on the management of CHA [12,13]. Studies have examined the effects of upper-segment cervical mobilization such as Mulligan SNAG and Maitland’s technique [14]. Our study is the first in which the Cyriax cervical mobilization technique is applied in CHA. There are studies showing the effectiveness of various exercise methods in CHA [15,16,17]. Pilates includes special movements intended to stabilize the joints, strengthen the deep spinal stabilizing muscles, lengthen the spine, increase mind–body awareness and improve posture [18]. The Australian Physiotherapy and Pilates Institute (APPI) has modified Pilates exercises so that they can be used in the clinic. This method aims to place five elements: lateral breathing, neutral spine position and centering, shoulder girdle placement, rib cage placement and head–neck placement [19]. In this context, the Pilates method appears to be an alternative for the treatment of musculoskeletal disorders [20]. It was reported that when individuals with CHA stay in a long-term fixed sitting position, postural compliance in the cervical, thoracic and lumbar spinal segments is lower than that of healthy individuals, and headache is triggered [21]. In light of the studies conducted, it is thought that a holistic exercise approach involving the entire spine, such as clinical Pilates, may be more effective in CHA instead of exercises that only concern the cervical region. There is a case series reporting that the Pilates exercise program can induce positive effects on disorders related to tension-type headaches [22]. However, there are no studies examining the effectiveness of clinical Pilates in CHA.

For the current study, we hypothesized that the combined use of cervical mobilization (CM) and clinical Pilates exercises (CPEs) is more effective than CM alone in patients with CHA. The present study aimed to examine the effects of CM and CM combined with CPE on pain frequency and intensity, the number of analgesics, cervical muscle stiffness and head and neck blood flow in individuals with CHA.

## 2. Materials and Methods

### 2.1. Study Design and Ethics

This study is a prospective and randomized controlled trial. The study was approved by the Clinical Research Ethics Committee of SANKO University in accordance with the Declaration of Helsinki (Session No: 2020/04, Decision No: 03). This study was registered at Clinicaltrials.gov (ID number: NCT05883319 (22 March 2022)).

### 2.2. Participants

**Figure 1:**
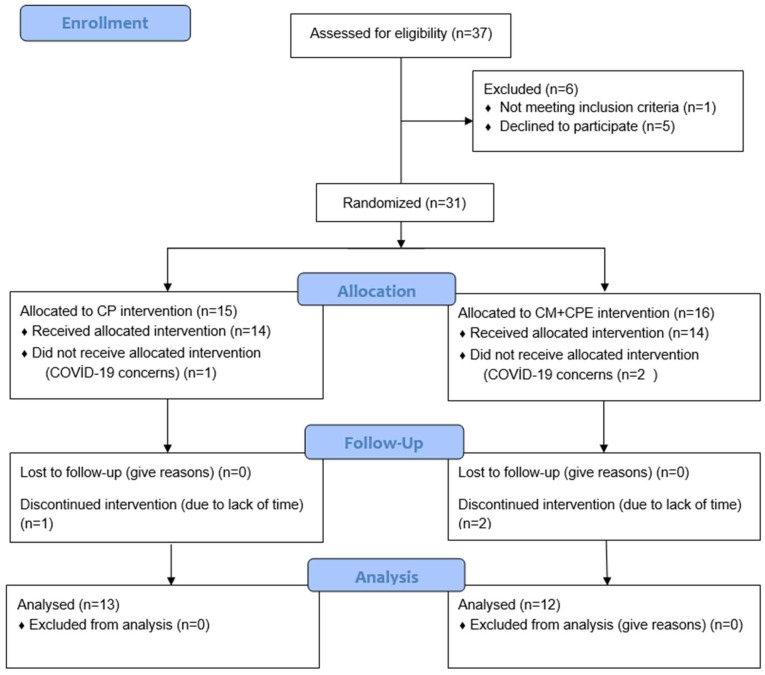
CONSORT Flow Diagram.

Consort flow diagram illustrating the participants in the study is presented in Figure 1. Patients who presented to the Neurology Outpatient Clinic of Sani Konukoğlu Practice and Research Hospital and were diagnosed with CHA by a specialist were included in the study. Individuals who signed the voluntary consent form and were included in the study were randomly divided into groups using the minimization method, considering their age, gender and presence of headache. Individuals were grouped into the CM and CM+CPE treatment groups.

The inclusion criteria were determined as follows: being diagnosed with CHA, being an adult between the ages of 18 and 65 and not having received medical (excluding analgesic) treatment or physiotherapy for CHA in the previous few months. The exclusion criteria were determined as follows: having undergone headache surgery, serious cardiac history or surgery, current or previous malignancy history and being diagnosed with epilepsy. We calculated the sample size based on power analysis (G*power version 3.1.9.2, Axel Buchner, Universität Kiel) with a large effect size of d: 0.8. With two-sided test (alpha = 5%, power = 95%), we estimated that based on the headache frequency variable, nine people were determined per group [23]. When post hoc power analysis was performed according to the difference in ICA blood flow within CM groups, the power of the study was found to be 0.97, with ICA blood flow within CM+CPE 0.18 (G*Power 3.1, Düsseldorf, Germany). Due to the possibility of data loss, it was planned to include 15 people in each group. In line with the inclusion criteria, 31 patients were included in the evaluation. Three out of six patients discontinued treatment due to COVID-19 concerns and the other three patients due to a lack of time. The study was finalized with a total of 25 patients, 12 in one group and 13 in the other group.

### 2.3. Measurements

While the head and neck blood flow evaluation was carried out by a radiologist with 20 years of experience, all other evaluations were performed face-to-face by the same physiotherapist under the same conditions. The evaluations were made before the treatment and after the treatment.

First, the demographic data of individuals such as gender, age, body weight (kg), height (cm) and body mass index (kg/m) and presence of headache (years) were also recorded.

Pain: headache frequency of pain (per month), headache intensity and analgesic use (number/month) were recorded. Headache intensity was evaluated in cm with visual analogue scale (VAS).

Muscle Stiffness: Suboccipital, upper trapezius and sternocleidomastoid (SCM) muscle stiffness of the patients was evaluated bilaterally with a myotonometer (Myoton AS, Tallinn, Estonia). Myotonometers are valid and reliable methods for assessing stiffness [24]. In muscle stiffness evaluations, SCM was evaluated in the supine position, suboccipital in the prone position and upper trapezius in the sitting position. The measurement was repeated 3 times for the right and left sides. The average of the measurements was taken and recorded in N/m unit [8,25].

Blood flow: A Siemens Acuson S2000 (Siemens, Erlangen, Germany) device was used to evaluate the patients’ internal carotid artery (ICA) and vertebral artery (VA) blood flow. Measurements were recorded for the right and left sides [26]. The average values of the right and left sides in blood flow were taken.

### 2.4. Interventions

All treatment methods were applied by the same physiotherapist under the same conditions, 3 times a week for 6 weeks. Before the treatment, the patients were informed about the treatment method applied.

#### 2.4.1. CM

Neck mobilization techniques of Cyriax were applied to one group. Prior to applying CM, the patients were placed in the supine position on the treatment bed. The vertebrobasilar artery (VBA) test was conducted before mobilization. All patients’ VBA tests were negative. The bridging technique, manual traction rotation with manual traction, anterior-posterior gliding with manual traction, lateral gliding and trapezoidal manual stretching were applied among CM techniques [27]. CM was applied for approximately 10 min as 8–10 repetitions for each technique.

#### 2.4.2. CPE

CPE program selected from the exercises developed by the APPI. The exercise program of all patients in the group was carried out in the presence of a physiotherapist certified as an exercise instructor by the APPI [19].

Firstly, the patients were shown the 5 basic elements of lateral breathing, neutral spine position and centering, shoulder girdle placement, rib cage placement and head–neck placement and taught them practically. During the exercise sessions, the patients were guided using verbal and tactile stimuli to ensure the continuity of these basic elements. Each exercise was performed with 8–10 repetitions in all sessions.

**Figure 2:**
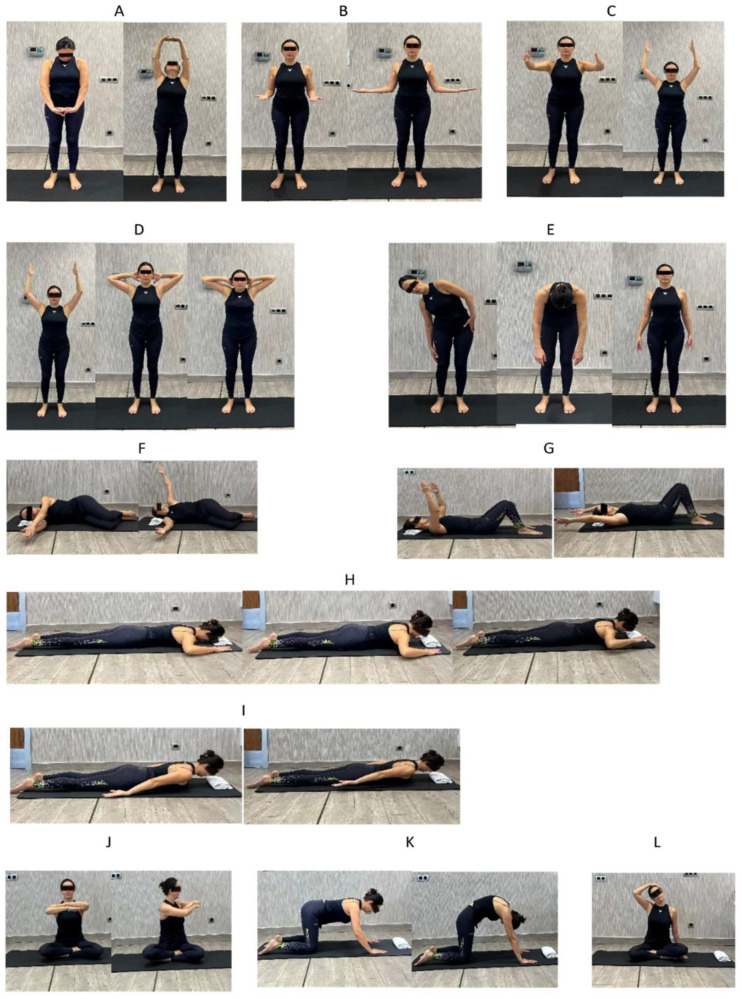
Clinical Pilates Programme. (**A**) Upper Back Warm-up; (**B**) Dumb Waiter; (**C**) Chest Stretch; (**D**) Corkscrew; (**E**) Upper Body Rolls; (**F**) Arm Opening Level 1; (**G**) Double Leg Stretch Level 1; (**H**) Swan Dive Level 1–3; (**I**) Breast Stroke Prep 1 Level 2; (**J**) Spine Twist; (**K**) Cat Stretch; (**L**) Active Trapezius Stretch.

The following exercise program was applied as shown in Figure 2. When planning the exercise program, it was aimed to increase DNFE, relax the muscles around the neck and increase spinal stability and mobility.

Only CM was applied to the 1st group, and CM and CPE were applied together to the 2nd group. Interventions in all groups were applied three days 3 times a week for 6 weeks.

### 2.5. Statistical Analysis

All analyses were conducted using SPSS software version 25. The normality and homogeneity of the data were evaluated with the Shapiro–Wilk test. As descriptive statistics, mean and standard deviation values were presented for continuous variables specified by the measurement, while frequency and percentage values were presented for qualitative variables. Categorical variables between the groups were compared using the chi-square test. The Mann–Whitney U test was used for comparisons between the groups. The pre-treatment and post-treatment values within groups were compared with the Wilcoxon signed rank test. The level of significance was taken as *p* < 0.05 for all analyses.

## 3. Results

In line with the inclusion criteria, 31 patients were included in the evaluation. Three out of six patients discontinued treatment due to COVID-19 concerns and the other three patients due to a lack of time. The study was finalized with a total of 25 patients, 13 in the CM group and 12 in the CM+CPE group. There was no difference between the groups in terms of age (*p* = 0.276), the presence of headache (*p* = 0.782) and gender (*p* = 0.748) (Table 1).

Pain frequency and intensity, the number of analgesics, muscle stiffness, blood flow values pre- and post-treatment and the change between the two groups are listed in Table 2. According to a within-group comparison, a decrease in pain parameters, suboccipital, upper trapezius and SCM muscle stiffness and an increase in ICA and VA blood flow were observed in both groups (*p* < 0.05).

According to a between-group comparison, pain intensity and left SCM muscle stiffness changes were found to decrease more significantly in the CM+CPE group compared to the CM group (respectively, *p* = 0.025, *p* = 0.044).

There was no difference in ICA and VA blood flow change between the two groups.

## 4. Discussion

The present study was carried out to compare the impacts of CM and CM+CPE on CHA and found decreased pain frequency and intensity, the number of analgesics and muscle stiffness and increased blood flow in two groups. However, CM+CPE was determined to be more effective in decreasing pain intensity and left SCM muscle stiffness.

### 4.1. Pain

The results of the present study demonstrated that CM alone and CPE+CM significantly decrease headache intensity, headache frequency and analgesic use in CHA. It is thought that CM relieves headache by reducing stress on cervical structures and increasing blood flow when applied alone or in combination with CPE.

Guidelines on CHA support the positive impacts of cervical mobilization and manipulation on headaches [28]. Furthermore, there are sources stating that therapeutic local exercises can be used in headache. A systematic review examining studies on patients with CHA showed that the use of craniocervical flexion, cervical stabilization, cervicoscapular strengthening, corrective exercises and active cervical region exercises might be effective [17]. CPE is an exercise approach based on neutral spine and core stabilization. In all exercises, neck stabilization is provided by deep neck flexor activation in the neck region, while scapular stabilization is ensured by activating the rhomboids, trapezius middle-lower part and serratus anterior in the shoulder girdle. In the meantime, the trapezius upper part and pectoral muscles are kept in a long position, and proper posture is tried to be maintained throughout the exercise period. There is no study in the literature in which CPE, an exercise approach that takes a holistic approach to the body and cares about the mind–body connection, is used specifically for headaches. Our study contributes to the literature in this respect. A significant improvement was observed in terms of headache frequency and intensity and the number of analgesics in both groups, which proves that the manual approach is effective in pain treatment. The addition of CPE to CM had a positive effect, especially on the decrease in pain intensity. There is a need for studies in which long-term results are monitored for the effect of CPE on headache.

### 4.2. Muscle Stiffness

It is known that CHA is caused by problems in the structures of the cervical region. Especially SCM, upper trapezius and suboccipital muscle tenderness and stiffness are frequently encountered [6,29,30].

A study examining the effects of dry needling and ischemic compression methods in the presence of SCM active trigger points associated with CHA reported that both methods were effective [31]. A study evaluated the effect of cervical stretching and craniocervical flexion exercises on cervical muscles in individuals with CHA and stated that craniocervical exercises were more effective in improving upper trapezius and suboccipital muscle stiffness measured with Myoton Pro [32].

Although there is serious evidence that tension and tenderness in the neck muscles affect CHA, there are very few studies in the literature on how interventions change muscle stiffness. In our study, the stiffness of these muscles decreased significantly in both groups. Left SCM stiffness decreased more in the group where CPE was applied. The present study contributes significantly to the literature in terms of showing that CM applied in CHA alone or in combination with CPE can reduce the stiffness of the neck muscles. Long-term follow-up studies are needed to determine how long the muscle-stiffness-reducing effect of CM and CPE lasts.

### 4.3. Blood Flow

There are recent studies in the literature, indicating the need to examine the relationship between headache and the vessels feeding the brain. How the applied treatment methods affect this blood flow has also become important.

In their study conducted with Doppler US, Abdullaiev R Ya et al. compared the VA blood flow of patients with CHA and healthy individuals and reported that blood flow decreased, especially in head rotation [10]. A study stated that VA blood flow increased significantly after acupressure application in a tension-type headache [33]. There is a study in the literature showing that VA blood flow increases significantly after neuromuscular joint facilitation in healthy individuals [34]. As a different opinion, another study examining VA and ICA blood flow after manipulation in individuals with chronic neck pain showed that blood flow parameters did not change [26]. It is stated that there is no change in head and neck blood flow after different head positions and cervical manipulation and that cervical interventions can be used safely [35]. Studies examining head and neck blood flow in the literature often question whether mobilization and manipulation interventions can be applied safely. Studies have been conducted to examine the effect of blood flow on chronic neck pain and different types of headaches. It is seen that there is no consensus among the studies showing different results. The study results showed that blood flow increased significantly in both groups. We believe that CM and CPE interventions can correct posture and relax circulation in individuals with CHA by reducing existing muscle tensions, thus increasing head and neck blood flow. Therefore, we think that more studies are needed. Our study will be the first in the literature to examine the effect of CHA on head and neck blood flow.

In the case report we presented earlier, we observed the acute effect of CM on headache intensity, blood flow, muscle stiffness and CROM. In line with the positive results we obtained, we stated that there was a need for randomized controlled trials on the subject [36]. In this randomized controlled study, we also observed the positive effects of CM after 6 weeks of treatment. Furthermore, we demonstrated the additional effect of CPE on pain and muscle stiffness by adding CPE to one group.

There are studies about the neck suggesting the use of CPE in patients with neck pain and sagittal cervical disorientation, but their number is very small [37]. Leite et al., in a case series, reported that the clinical Pilates program can induce positive effects on pain intensity, daily life function and negative emotional states related to tension-type headaches [22]. Studies examining the effect of CPE on headaches are quite limited. A study concluded that CPE was an effective and safe exercise for pain, deep neck flexor, posture, the cervical range of motion and proprioception in patients with non-specific chronic neck pain [38]. Our study showed that CPE contributed to the decrease in headache intensity and muscle stiffness in CHA, which is a headache originating from the neck.

## 5. Conclusions

It is impossible to ignore the increase in neck and cervical disorders due to modern lifestyles and the use of technological devices, and cervicogenic headache is one of these disorders with an increasing incidence. The increase in the use of analgesics, especially for chronic musculoskeletal pain, represents a significant burden on the healthcare system. In addition, given the negative effects of long-term analgesic use, it is important to develop alternative approaches to pain relief in terms of human health. Our study contributes to the literature by investigating the effects of CM and CPE in combination with CM on pain, muscle stiffness and cervical blood flow in CHA and offers alternative solutions. Studies on the long-term effects of specific exercise programs and manipulative treatment approaches applied to CHA are needed.

In summary, alternative approaches in the management of musculoskeletal pain are important for the health systems of countries. In patients with CHA, CM was found to reduce headache intensity, headache frequency, the number of analgesics used and neck muscle stiffness and increase neck blood flow. It was concluded that CM and CPE together may be more effective in reducing pain intensity and muscle stiffness. It was suggested that it would be beneficial to add these interventions to the treatment plans of patients with CHA.

The limitation of our study is that there was no follow-up to check the sustainability of the effects of the interventions.

## Figures and Tables

**Table 1:** Demographics and presence of headache.

	CM (n = 13)	CM+CPE (n = 12)	*p*
	X ± SD	X ± SD	
Age (years)	34.9 ± 9.214	30.25 ± 9.016	0.276 ^b^
Presence of headache (years)	3.38 ± 2.142	3.25 ± 2.454	0.782 ^b^
BMI (kg/m^2^)	21.432 ± 4.896	20.612 ± 3.946	0.744 ^b^
Gender	n (%)	n (%)	
Famele	9 (69)	9 (75)	0.748 ^a^
Male	4 (31)	3 (25)	

X Mean, SD Standard deviation, BMI Body mass index, CM Cervical mobilization group, CPE Clinical Pilates exercise group; ^a^ Chi-square; ^b^ Mann–Whitney U test.

**Table 2:** Comparisons of outcome measures within and between groups.

			CM X ± SS	*p* ^a^	CM+CPE	*p* ^a^	Z	*p* ^b^
Pain	Frequency (times month)	Pre-treatment	13.38 ± 7.54	0.001 *	17.08 ± 10.71	0.002 *		
		Post-treatment	3.62 ± 2.78		3.58 ± 2.99			
		Change	9.769 ± 5.433		13.5 ± 8.969		−0.982	0.325
	Intensity (cm)	Pre-treatment	5.81 ± 1.18	0.001 *	7.43 ± 1.04	0.002 *		
		Post-treatment	1.74 ± 1.41		1.88 ± 1.14			
		Change	4.072 ± 1.076		5.55 ± 1.720		−2.232	0.025 *
	Number of analgesics (per month)	Pre-treatment	12.23 ± 10.94	0.001 *	13.75 ± 12.70	0.002 *		
		Post-treatment	2.62 ± 3.30		2.42 ± 2.39			
		Change	9.615 ± 8.713		11.33 ± 11.364		−0.491	0.623
Muscle stiffness (N/m)	R suboccipital	Pre-treatment	331.85 ± 98.75	0.001 *	304.92 ± 70.98	0.002 *		
		Post-treatment	254.23 ± 67.46		232.08 ± 54.45			
		Change	77.61 ± 85.81		72.83 ± 44.29		−0.598	0.549
	L suboccipital	Pre-treatment	326.69 ± 98.78	0.001 *	302.83 ± 76.13	0.002 *		
		Post-treatment	237.69 ± 45.17		236 ± 47.94			
		Change	89 ± 94.36		66.83 ± 46.99		−0.163	0.870
	R upper trapez	Pre-treatment	291.08 ± 47.40	0.001 *	319.42 ± 55.64	0.002 *		
		Post-treatment	243.62 ± 40.39		268.58 ± 51.25			
		Change	47.461 ± 26.38		50.83 ± 17.23		−1.008	0.313
	L upper trapez	Pre-treatment	299.08 ± 53.87	0.001 *	332.42 ± 68.32	0.002 *		
		Post-treatment	255.46 ± 46.01		275.50 ± 54.20			
		Change	43.61 ± 30.30		56.58 ± 27.87		−1.142	0.253
	R SCM	Pre-treatment	242.69 ± 45.31	0.001 *	246.50 ± 45.83	0.002 *		
		Post-treatment	210.08 ± 22.29		204.83 ± 28.02			
		Change	34.30 ± 36.88		41.66 ± 21.70		−1.715	0.086
	L SCM	Pre-treatment	233.38 ± 21.9	0.001 *	253.25 ± 58.77	0.002 *		
		Post-treatment	210.08 ± 22.29		209.92 ± 52.15			
		Change	23.307 ± 12.31		43.33 ± 29.61		−2.013	0.044 *
Blood flow (cm/s)	ICA PS	Pre-treatment	111.36 ± 14.86	0.001 *	118.89 ± 13.11	0.002 *		
		Post-treatment	112.62 ± 14.95		120.64 ± 13.22			
		Change	1.261 ± 1.025		1.745 ± 1.104		−1.198	0.231
	ICA ED	Pre-treatment	37.62 ± 7.63	0.011 *	40.31 ± 4.38	0.005 *		
		Post-treatment	38.46 ± 7.27		42.40 ± 5.462			
		Change	0.842 ± 0.902		2.087 ± 3.207		−1.306	0.191
	VA PS	Pre-treatment	82.75 ± 16.35	0.034 *	70.720 ± 15.41	0.010 *		
		Post-treatment	83.39 ± 16.72		71.49 ± 15.33			
		Change	0.638 ± 0.975		0.775 ± 0.907		−0.326	0.744
	VA ED	Pre-treatment	27.43 ± 6.80	0.012 *	25.85 ± 6.50	0.015 *		
		Post-treatment	28.05 ± 6.86		27.01 ± 6.40			
		Change	0.623 ± 0.709		1.160 ± 1.382		−0.708	0.479

Mean ± Standard deviation (SD). * *p* < 0.05 for *p* ^a^ (within the group) by Wilcoxon test and *p* ^b^ (between groups) by Mann–Whitney U test. CM cervical mobilization group, CPE clinical Pilates exercise group, R right, L left, SCM sternocleidomastoideus, ICA internal carotid artery, VA vertebral artery, PS peak systolic, ED end diastolic.

## Data Availability

The data presented in this study are available on request from the corresponding author. The data are not publicly available due to privacy.

## Abbreviations

CHA, cervicogenic headache; APPI, the Australian Physiotherapy and Pilates Institute; CM, cervical mobilization; CPE, clinical Pilates exercise; VAS, visual analogue scale; SCM, sternocleidomastoid; ICA, internal carotid artery; VA, vertebral artery; X, mean; SD, standard deviation; BMI, body mass index; R, right; L, left; PS, peak systolic; ED, end diastolic.
